# Screening apelin analogues and a small molecule agonist as effective cardiovascular therapeutics against reperfusion injury

**DOI:** 10.1039/d5md00985e

**Published:** 2025-12-19

**Authors:** Anjalee Wijewardane, Kleinberg X. Fernandez, Anran Zhang, Conrad Fischer, Dineth P. Nagahawatta, Xavier Iturrioz, Charlotte Avet, Bethan L. Heard, Catherine Llorens-Cortes, Gavin Y. Oudit, Michel Bouvier, John C. Vederas

**Affiliations:** a Department of Chemistry, University of Alberta Edmonton AB T6G 2G2 Canada john.vederas@ualberta.ca; b Department of Physiology, University of Alberta 8440-112 Street NW Edmonton Alberta T6G 2B7 Canada; c Mazankowski Alberta Heart Institute, University of Alberta, 8440-112 St. NW Edmonton Alberta T6G 2B7 Canada; d Center for Interdisciplinary Research in Biology (CIRB), College de France, INSERM, CNRS Paris F-75005 France; e Département Médicaments et Technologies pour la Santé, SIMoS, CNRS ERL9004 Gif-sur-Yvette 91191 France; f Institute for Research in Immunology and Cancer (IRIC), Department of Biochemistry and Molecular Medicine, Université de Montréal Montréal QC H3T 1J4 Canada

## Abstract

Ischemic reperfusion injury is a major global health threat, with the development of effective therapeutics still urgently needed. While both peptide therapeutics and small-molecule agonists have been extensively investigated to treat this condition, in this study, we explored the pharmacological potential of retro-inverso apelin peptides, including parent and modified forms, by evaluating their binding activity and physiological effects. We further assessed two metabolically stable apelin peptide analogues previously developed in our lab and compared them to a small-molecule agonist, BMS-986224, for biased agonist properties. Using radioligand displacement assays and blood pressure assays, we found that the retro-inverso peptides lacked apelin receptor binding affinity and physiological activity. However, our metabolically stable analogues and the small molecule demonstrated good receptor binding. Notably, we showed the metabolically stable apelin-13 analogue, NMeLeu13A2, to exhibit little to no blood pressure-lowering effects but retain cardiorestorative effects in the Langendorff assay. In contrast, CbzPEG_6_–NMeLeu17A2 not only provided cardioprotective effects but also significantly lowered blood pressure. These findings highlight the potential of NMeLeu13A2 for targeted therapeutics and underscore the promise of apelin-based analogues in addressing ischemic reperfusion injury.

## Abstract 2

In this study, we investigate the cardioprotective effects of metabolically stable agonists targeting the apelin receptor using relevant biochemical assays.

## Graphical abstract

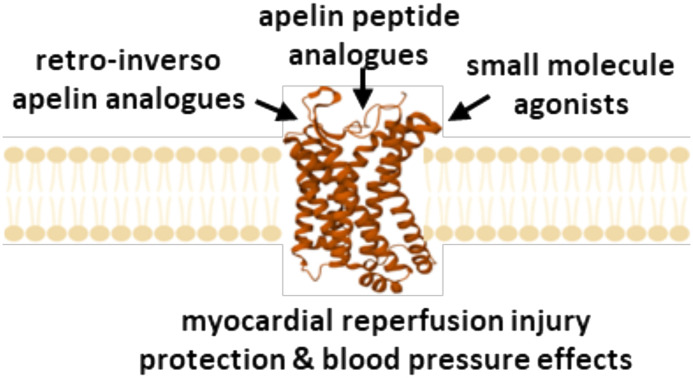

## Introduction

Coronary heart disease is a leading cause of death worldwide and places a substantial financial burden on healthcare systems.^1^ This condition often involves the build-up of cholesterol plaques in coronary arteries, which can lead to a heart attack, medically termed myocardial infarction.^2^ Treatment to restore blood flow is essential. However, the sudden reintroduction of oxygenated blood can exacerbate tissue damage and activate pathways that cause heart cell necrosis.^3^ This myocardial ischemia–reperfusion injury (MIRI), is a target for development of new treatments.

The apelinergic system is a regulator in cardiovascular function and has shown positive effects on myocardial contractility, vasodilation,^4,5^ angiogenesis^6^ and heart rate.^7^ Apelin promotes vasodilation by stimulating nitric oxide (NO) production, with apelin potentiating phosphorylation of Akt and eNOS,^5,8^ while its signalling through Akt and ERK pathways also helps regulate cardiac contractility and provides protection against cardiac hypertrophy.^9^ Apelin peptide hormones are derived from a 77-residue pre-propeptide and exist as three major isoforms, differentiated by their length, (pyr)^1^-apelin-13, apelin-17, and apelin-36^10^ that bind to the apelin receptor (apelinR). However, the short half-life of these peptides limits their use as therapeutics to treat MIRI.^4,11^ In our previous work, we studied the degradation of the apelin-13 and apelin-17 isoforms by enzymes such as angiotensin-converting enzyme 2 (ACE-2),^11^ neprilysin (NEP),^12^ and plasma kallikrein (KLKB1),^13^ and introduced modified amino acid residues at cleavage sites to improve the half-lives of apelin peptides.^14,15^ Our most potent analogue, CbzPEG_6_–NMeLeu17A2, is an apelinR agonist that is stable in blood (*t*_1/2_ > 18 h in human plasma), lowers blood pressure (BP), and shows high potency in the Langendorff heart assay.^14^ Another peptide analogue we designed previously and investigate further in this study is NMeLeu13A2, which has a half-life of ∼4 hours in human plasma.^15^ We showed previously that it didn't lower blood pressure and hence did not study its cardioprotective effects further at that time. However, both analogues are not orally bioactive and require administration by injection. Backbone-modified apelin analogues have been reported including polyethylenglycol (PEG) backbone exchange and ring-backbone cyclization leading to active^16,17^ and activity-compromised peptide agonists.^18,19^ An alternative approach to developing metabolically resistant peptides is the use of retro-inverso (RI) peptides. Such analogues consist of d-amino acids arranged in reverse order with inverted amide bonds, maintaining similar side chain superimposition to their natural counterparts when extended.^20,21^ These peptides are particularly effective when side chain interactions dominate over backbone recognition, and they have shown promise in some anticancer applications.^20,22^ Studies of analogues of angiotensin I and bradykinin demonstrated that corresponding retro-inverso peptides can be metabolically stable cardiovascular regulators.^23^ Two other successful examples involving GPCRs include a heptapeptide retro-inverso inhibitor that reversed angiotensin receptor autoantibody-induced hypertension,^24^ and prosaptide D5, an 11-mer retro-inverso peptide that reversed hyperalgesia in rats by inhibiting voltage-gated calcium channels through a GPCR-mediated pathway.^25^

If successful as receptor binders, d-peptides can offer enhanced resistance to proteolytic degradation, prolonged half-life, potentially low cytotoxicity and improved oral bioavailability.^21^

Another avenue being investigated is the use of small molecule as agonists for the apelinR, such as, Bristol Myers Squibb's BMS-986224,^26,27^ Amgen's AMG986,^28^ and Davenport's CMF-019.^29^ Among these compounds, BMS-986224 was identified as a selective small molecule agonist that displays positive cardiac effects without significantly lowering blood pressure in a rat pressure–volume loop model.^26^

In this study, two approaches to metabolically more stable apelin analogues were investigated. In the first, four retro-inverso (RI) apelin peptide analogues were examined as possible agonists for the apelinR (Table 1). Two correspond to the native apelin-13 and apelin-17 whereas two others are based on previous active analogues, NMeLeu13A2 and NMeLeu17A2.^30^ In the second approach, we re-investigate the cardioprotective potency of NMeLeu13A2, using BMS-986624 as a standard, and compare our best analogue, CbzPEG_6_–NMeLeu17A2, through physiological assays and signalling profiles for G-protein activation and β-arrestin recruitment using bioluminescence resonance energy transfer (BRET) assays.

**Table 1:** Apelin peptide analogues investigated in this study

Compound	Sequence
pyr-apelin-13 (native)	pE-RPRLSHKGPMPF
Apelin-17 (native)	KFRRQRPRLSHKGPMPF
	
NMeLeu13A2	pE-RPRNMeL-SHKGP-Nle-Aib-BrF
CbzPEG_6_–NMe17A2	Cbz-PEG_6_-KFRRQRPRNMeL-SHKGP-Nle-Aib-BrF
	
RI-13	fpmpGkhslrprq
RI-13A2	Brf-Aib-nle-pmpGkhslrprq
RI-17	fpmpGkhslrprqrrfk
RI-17A2	Brf-Aib-nle-pmpGkhslrprqrrfk
C-terminal 8-mer	SHKGPMPF
C-terminal 5-mer	GPMPF
N-terminal 9-mer	KFRRQRPRL

## Results and discussion

### Testing retro-inverso (RI) peptide analogues for receptor binding activity and blood-pressure (BP) lowering activity

RI peptides were synthesized C→N terminally using solid phase peptide synthesis (SPPS, SI). RI-13A2 and RI-17A2 (Table 1) incorporate unnatural d-amino acid isosteres Brf, Aib, nle as the corresponding l-amino acid versions were used to stabilize the N-terminus against ACE-2.^9^ The newly synthesized peptides (Table 1) were tested for activity in two assays. First, their ability to compete with the radioligand [^125^I]-(Nle^11^, Tyr^13^) apelin-13 (0.2 nM) was compared to the references, native pyr-apelin-13 and apelin-17. Second, the physiological activity of the RI peptides was assessed using a BP lowering assay against the positive control, CbzPEG_6_–NMeLeu17A2. As shown in Fig. S2, all the RI peptides exhibited little to no binding to the apelinR. Consequently, no blood pressure-lowering effect was observed, as expected (Fig. S3). Although evidence from structural studies, including recent cryo-EM and AlphaFold2 models,^31–33^ highlight the significance of side-chain interactions—especially those in the orthosteric site— for ligand recognition and receptor activation, the lack of binding of the RI peptides to the apelinR in our experiments suggests that apelin–apelinR binding depends not only on specific side-chain interactions but also critically on the amide backbone structure.

### Comparison of receptor binding of apelin peptide analogues *vs.* the small molecule agonist BMS-986224

To evaluate the binding affinities of apelin peptide analogues compared to small-molecule agonists, we focused on BMS-986224, which was reported to have a *K*_d_ of 0.3 nmol L^−1^ (nM).^27^

**Fig. 1:**
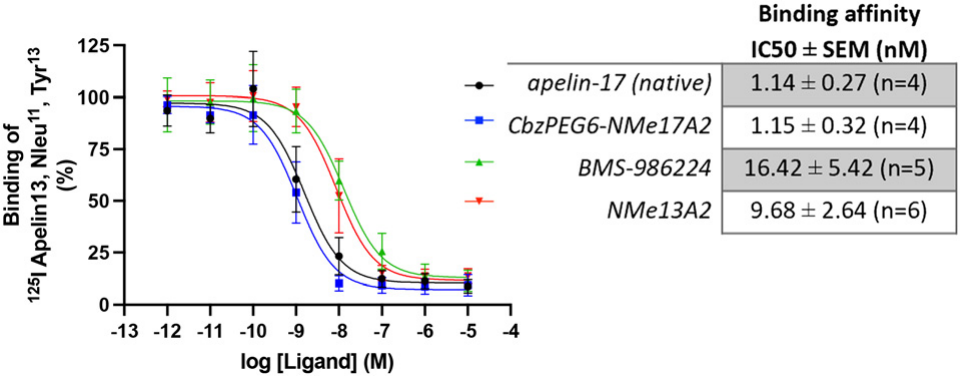
Competition binding curves of different apelin receptor ligands. Membranes of CHO cells stably expressing the hApelinR were incubated with 0.2 nM of radiolabeled [^125^I]-apelin-13 (apelin 13, Nle^11^, Tyr^13^) in the presence of increasing concentrations of each ligand (from 1 pM to 10 μM). Data are expressed as a percentage of maximal binding of [^125^I]-apelin-13 in the absence of any unlabeled ligand. Graphic is the compilation of *n* independent experiments performed in duplicate (*n* from 4 to 6). Error bars represent the SD of 4 to 6 independent experiments.

For comparison, we selected our most metabolically stable peptide analogues from each isoform, CbzPEG_6_–NMeLeu17A2 and NMeLeu13A2 and evaluated their binding affinities using BMS-986224 as a standard. All tested agonists displayed an affinity for the apelinR in the nanomolar range. The apelin-17 isoforms, native apelin-17 (1.14 ± 0.27 nM) and CbzPEG_6_–NMeLeu17A2 (1.15 ± 0.32 nM), showed higher binding affinities compared to the apelin-13 analogue, NMeLeu13A2 (9.68 ± 2.64 nM, Fig. 1). Among the tested compounds, the small-molecule agonist BMS-986224 showed the weakest binding (16.42 ± 5.42 nM) in our studies.

### Evaluating apelin peptide analogues and small molecule agonist BMS-986224 on blood pressure (BP) lowering activity

The metabolically stable apelin analogues from each isoform, CbzPEG_6_–NMeLeu17A2 and NMeLeu13A2 along with the small molecule agonist, BMS-986224 (0.5 μmol kg^−1^ infusion during 5 min for each compound), were tested for their BP lowering activity in anesthetized wild-type male mice (C57BL/6, 24–30 g). Among the compounds tested, only CbzPEG_6_–NMeLeu17A2 demonstrated a significant hypotensive effect (Fig. S3). The apelin-13 analogue, NMeLeu13A2, did not significantly alter BP and had a limited impact on hemodynamics. Similarly, BMS-986224 showed no hypotensive effect during the experiment compared to the vehicle control. In contrast, the hypotensive effect of CbzPEG_6_–NMeLeu17A2 was observed immediately after infusion. This analogue reduced mean arterial pressure (MAP) by approximately 50% during the experiment, with the effect sustained for more than 30 minutes post-administration. Apelin can act on the endothelial layer of vasculature, leading to systemic changes in blood pressure by increasing the synthesis of nitric oxide (NO).^5^ The effect of CbzPEG_6_–NMeLeu17A2 on vasodilation may be attributed to its hypotensive effect, which decreases the preferential resistance by increasing eNOS activation and NO production through the activation of the PI3K/Akt signalling pathway on endothelial cells like other apelin analogues we tested in the previous studies.^5,11^

### Evaluating apelin peptide analogues and small molecule agonist BMS-986224 in the Langendorff heart perfusion protocol

**Fig. 2:**
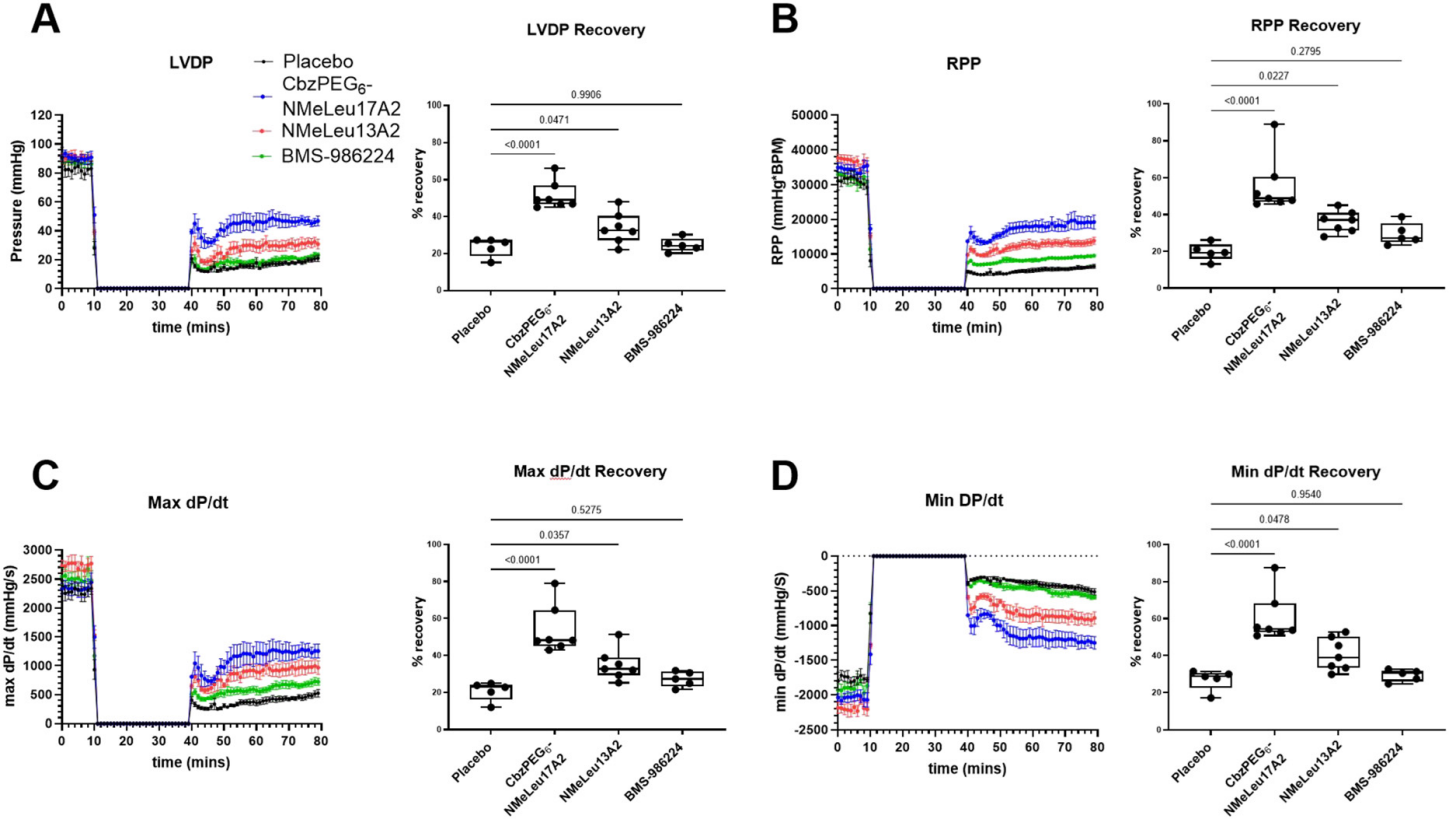
Myocardial ischemic reperfusion injury protection from apelin analogues and small molecule agonist. A) Left ventricular developed pressure (LVDP), B) rate pressure product (RPP), C) maximum (max d*P*/d*t*), and D) the minimum (min d*P*/d*t*) rate change in left ventricle pressure demonstrated the protective effect of 1 μmol L^−1^ of CbzPEG_6_–NMeLeu17A2 (*n* = 7), NMeLeu13A2 (*n* = 7), and BMS-986224 (*n* = 5) at the concentration of 1 μM, compared to placebo (vehicle, *n* = 5). All groups underwent ischemia from the 10th to the 40th minute of the experiment, as indicated in the figure. The placebo group received no treatment and was solely perfused with saline as the control. The recovery rate was calculated based on the percentage ratio between the average readings during the last 10 minutes of reperfusion and the average baseline readings. The treatment was administered at 1 μM. Values are expressed as mean ± SEM, and the significance level has been indicated as a number compared to the placebo using one-way ANOVA followed by Dunnett's test.

We also used the *ex vivo* Langendorff heart perfusion with ischemia protocol to test the difference between the BMS-986224, NMeLeu13A2, and CbzPEG_6_–NMeLeu17A2 in ischemia–reperfusion damage. After conditioning with 30 minutes of ischemia, 1 μM of the treatment was added to the perfusion buffer for 10 minutes, followed by perfusion with no treatment for 30 minutes. CbzPEG_6_–NMeLeu17A2 and NMeLeu13A2 showed a significant cardioprotective effect and positive inotropic effect for the global ischemia–reperfusion injury (Fig. 2.A–D) in left ventricle-developed pressure (LVDP) (Fig. 2.A), rate pressure product (RPP) (Fig. 2.B), systolic (max d*P*/d*t*) (Fig. 2.C), and diastolic functions (min d*P*/d*t*) (Fig. 2.D). CbzPEG_6_–NMeLeu17A2 showed a trend that is superior to NMeLeu13A2 in cardiac protection. CbzPEG_6_–NMeLeu17A2 nearly doubled the cardiac recovery compared to the placebo treatment in terms of left ventricle contractility, and diastolic and systolic function. NMeLeu13A2 showed a significant increase in recovery over the placebo but less than CbzPEG_6_–NMeLeu17A2, which differed from the BMS-986224, which failed to show significance in cardiac function recovery after the ischemia–reperfusion injury at 1 μmol L^−1^. The difference between the three agonists may be attributed to their varying binding affinities to the apelinR. Activation of the apelinR can promote the phosphorylation of the extracellular signal-regulated kinase (ERK) pathway, thereby enhancing cell survival in ischemia–reperfusion injury.^2^ The effect of activating ERK can be achieved through the downstream signaling pathways of β-arrestin or Gαi/o. However, β-arrestin signaling may promote receptor internalization, leading to desensitization of the signal.^32^ Additionally, β-arrestin-dependent ERK activation and/or increased nitric oxide production *via* eNOS can cause a decrease in blood pressure, rather than the Gαi/o-dependent pathway.^34^ This may explain why NMeLeu13A2 differs from CbzPEG_6_–NMeLeu17A2 in the blood pressure assay but is similar in reperfusion recovery. Apelin also has a positive inotropic effect, increasing Ca^2+^ availability to enhance heart contractility by activating the sarcolemma Na^+^/H^+^ exchanger with the increase in intracellular pH.^35^ Additionally, the increase in intracellular Ca^2+^ may be correlated with the activation of phospholipase C (PLC) and protein kinase C (PKC) as well.^36^

### Studying signaling bias of apelin peptide analogues and small molecule agonist BMS-986224 using bioluminescence resonance energy transfer (BRET) assays

**Fig. 3:**
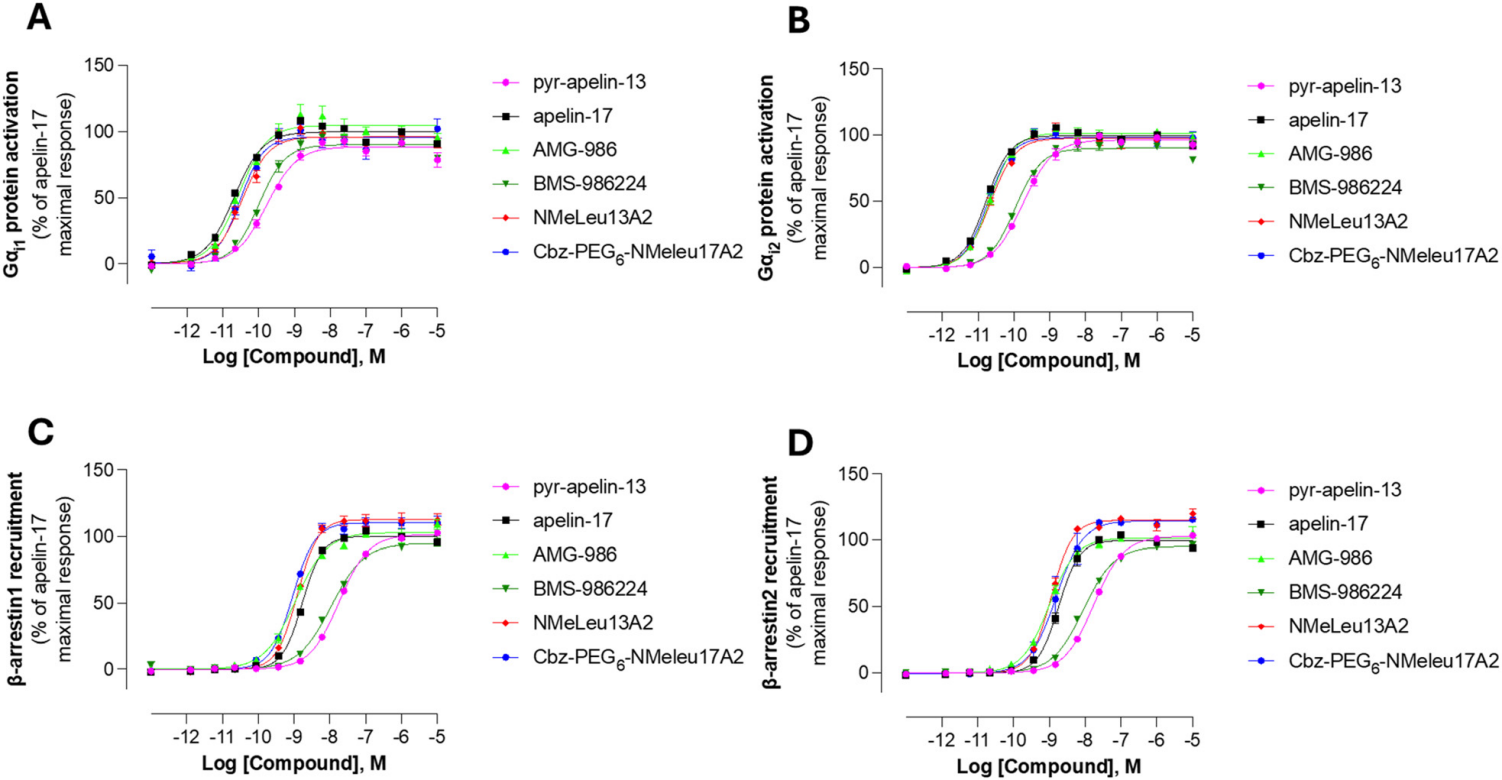
Concentration–response curves of endogenous apelin peptides (apelin-17 and pyr-apelin-13) and investigated analogs on human (A) Gαi1 activation, (B) Gαi2 activation, (C) β-arrestin1 recruitment, and (D) β-arrestin2 recruitment. The data represent the mean ± the SEM of 5 to 6 independent experiments performed in simplicate. The data are expressed as percentages of the maximal response obtained for apelin-17. log EC_50_ values are listed in Table S1.

The therapeutic potential of biased and unbiased agonists for the apelinR remains an area of active investigation, particularly regarding their effects on BP regulation. Previous studies have shown that BP changes induced by modified apelin-17 analogues may be due to Gαi-independent but β-arrestin-dependent signaling pathways.^34,37^ In contrast, studies on G-protein-biased agonists, such as CMF-019 and MM07, have demonstrated vasodilation, suggesting that BP lowering effects can occur without β-arrestin signaling engagement.^17,29^ In contrast, the two non peptidic apelinR agonists, BMS-986224 and AM-812337 as well as the metabolically resistant apelin-17 analog LIT01-196^38^ do not induce any change in BP in an myocardial infarction (MI) rat model and in an hypertension-induced cardiac dysfunction rat model.^26^ However, in anesthetized normotensive rats, there is a slight but significant decrease in BP at the beginning of the infusion with the BMS-986224, whereas LIT01-196 given by intravenous route in alert normotensive or hypertensive rats induce a profound decrease in BP.^39^ The effects of AM-8123 or AMG-986 on BP in normotensive animals were not reported. Due to these various behaviors of apelinR agonists, we evaluated the signaling profiles of our lead compounds, CbzPEG_6_–NMeLeu17A2 and NMeLeu13A2, alongside BMS-986224 and AMG-986. As illustrated in Fig. 3, the small molecule BMS-986224 shows close signaling resemblance to native apelin-13, generally displaying up to 1 order of magnitude lower efficacy than the other investigated compounds. Despite differences in their ability to lower BP, none of the compounds tested exhibited signaling bias, discounting ligand bias as sole cause for the observed BP reduction of some agonists. Previous structural studies have shown that small molecules mimicking the C-terminal Phe bind to a single orthosteric site, whereas apelin peptides bind to two orthosteric sites.^33^ To investigate whether this dual-site binding promotes different signaling biases, we synthesized and tested apelin C-terminal and N-terminal truncations (Table 1, C-terminal 5/8-mer and N-terminal 9-mer). However, we observed no activation of either Gi or β-arrestin, likely due to poor binding to the apelinR receptor (Table S1).

### Activation of Akt and ERK1/2 signaling pathways

**Fig. 4:**
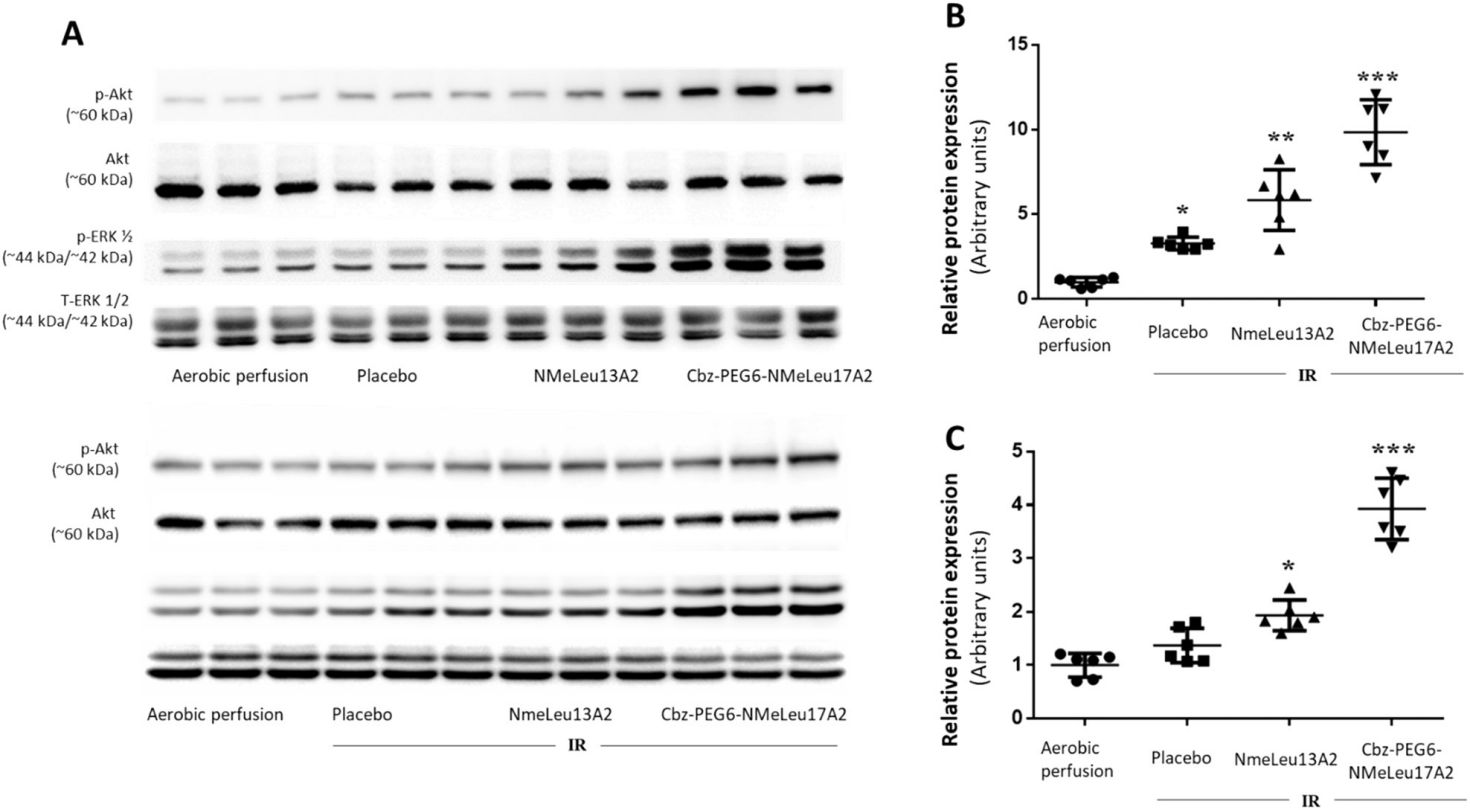
Phosphorylation of Akt and ERK1/2 in left ventricular tissue following ischemia–reperfusion (IR) and treatment with apelin analogues. Protein lysates were analyzed by Western blotting and band intensities quantified by densitometry. (A) Representative Western blots for phosphorylated Akt (p-Akt, Ser473), total Akt, phosphorylated ERK1/2 (p-ERK1/2, Thr202/Tyr204), and total ERK1/2. (B) Quantification of p-Akt/Akt. (C) Quantification of p-ERK1/2/ERK1/2. Data are expressed in arbitrary units relative to aerobic perfusion (control) and presented as mean ± SEM (*n* = 6 per group). Statistical analysis was performed using one-way ANOVA with Tukey's multiple comparisons test. Symbol key: *p < 0.05 *vs.* aerobic perfusion; **p < 0.05 *vs.* aerobic perfusion and placebo; ***p < 0.05 *vs.* all other groups.

Phosphorylation of Akt and ERK1/2 in left ventricular tissue was evaluated following ischemia–reperfusion (IR) and treatment with apelin analogues (Fig. 4). Treatment with NMeLeu13A2 and CbzPEG_6_–NMeLeu17A2 further enhanced Akt phosphorylation relative to control (mean differences −4.839 and −8.857; both *p* < 0.0001). Compared with IR, NMeLeu13A2 produced a modest but significant increase (*p* < 0.05), whereas CbzPEG_6_–NMeLeu17A2 elicited a robust enhancement (*p* < 0.0001). Notably, CbzPEG_6_–NMeLeu17A2 induced significantly greater Akt activation than NMeLeu13A2 (mean difference −4.018, *p* < 0.001). Meanwhile, both NMeLeu13A2 and CbzPEG_6_–NMeLeu17A2 significantly increased ERK1/2 phosphorylation compared with control (*p* < 0.01 and *p* < 0.0001, respectively). CbzPEG_6_–NMeLeu17A2 elicited a significantly stronger effect than NMeLeu13A2 (mean difference −1.991; *p* < 0.0001). Relative to IR, only CbzPEG_6_–NMeLeu17A2 produced a significant increase in ERK1/2 activation (*p* < 0.0001). Overall, both apelin analogues enhanced phosphorylation of Akt and ERK1/2, with CbzPEG_6_–NMeLeu17A2 exerting the most pronounced activation of prosurvival signaling pathways. The difference in hypotensive effects between NMeLeu13A2 and CbzPEG_6_–NMeLeu17A2 remains to be clarified and may involve mechanisms beyond eNOS, ERK1/2, and AKT activation. Since the BP assays were performed *in vivo*, we cannot exclude the possibility that these compounds differentially affect other systems such as the neuronal network or the kidney, both of which play key roles in BP regulation.^40,41^ It is important to note that the BRET assays, which showed similar receptor-G protein coupling and β-arrestin recruitment for NMeLeu13A2 and CbzPEG_6_–NMeLeu17A2, resulting in no signaling bias, were performed in HEK293 cells and therefore do not reflect the downstream vascular signaling environment. These assays cannot fully capture activation of later effectors such as ERK1/2, Akt, PI3K, or ultimately eNOS, which drives NO-mediated vasodilation.^5,34^ Because eNOS/NO represents the dominant pathway for apelin's hypotensive effects, the absence of BP lowering by NMeLeu13A2 may simply reflect that the activation of this signaling arm by this compound is not sufficient to cause BP decrease, despite preserved receptor engagement.

## Conclusions

The development of metabolically stable apelin agonists with cardioprotective, yet blood pressure neutral effect is of significant interest for addressing ischemic reperfusion injury. The first part of this study examined the relevance of RI peptides derived from native and modified apelin analogues of the 13 and 17 isoforms. Little to no receptor binding activity or physiological effects were observed with these peptides at the apelinR. However, these findings highlight the critical role of the backbone arrangement in receptor binding, showing that side-chain interactions and conformations alone are insufficient. The second part of this study compared previously developed apelin peptide analogues with small-molecule agonists. The signaling profiles showed no activation pathway bias for any of the apelinR agonists tested. Based on combined BP and Langendorff data, neither of the retro-inverso peptides nor the small agonist molecule (BMS-986224) demonstrated a hypotensive effect or protective role in ischemia–reperfusion injury. Instead, we identified two apelin analogues, CbzPEG_6_–NMeLeu17A2 and NMeLeu13A2, the first one more potent than the second in attenuating cardiac damage caused by ischemia–reperfusion. While CbzPEG_6_–NMeLeu17A2 was active in both the BP and Langendorff assays, NMeLeu13A2 was active only in the Langendorff assay at the studied dosage regime. To probe whether this difference in physiological effect was due to biased signaling, we performed BRET assays, which showed similar activation of Gαi1/2 and β-arrestin1/2 across ligands. We next examined ERK and Akt activation in the left ventricle, which showed a more pronounced response for CbzPEG_6_–NMeLeu17A2. Thus, such biased activity may also occur in endothelial cells on eNOs phosphorylation and elicit an impaired hypotensive activity of NMeLeu13A2 compared to CbzPEG_6_–NMeLeu17A2. However, as discussed earlier, the lowering of BP may involve complex mechanisms that are not yet fully understood. Although further studies are needed and are beyond the scope of the present work, the observation that NMeLeu13A2 enhances cardiac output without reducing BP suggests potential therapeutic value in settings where BP lowering is not desired or could be contraindicated. This makes it a promising candidate for the development of targeted therapeutics. Future efforts will focus on creating more stable analogues to enhance oral bioavailability, using NMeLeu13A2 as a lead compound for effective cardioprotective therapeutics.

### Synthetic procedures

#### General procedure for synthesis and purification of RI apelin analogues

All four retro-inverso peptides investigated, listed in Table 1, were synthesized using solid phase peptide synthesis (SPPS). For loading, 2-chlorotrityl chloride resin was transferred to a SPPS vessel and washed with dry DCM (3 × 5 mL) and then dry DMF (3 × 5 mL) for one min each, and then bubbled under argon in dry DMF (5 mL) for 10 min. The first Fmoc-protected amino (1.0 equiv. to desired resin loading) and DIPEA (5.0 equiv.) was mixed in 10 mL of DCM : DMF (1 : 1) and was bubbled under argon for 2.5 h, with continuous addition of DCM to top-up volume. Dry MeOH (0.8 mL g^−1^) was added to the resin and bubbled under argon for 15 min to end-cap remaining chlorotrityl groups. Post end-capping, the resin was washed as mentioned previously. Fmoc-deprotection was performed by bubbling 20% piperidine in DMF (3 × 5 mL) for 5 min each. For peptide elongation, the subsequent Fmoc-protected amino acid (1.1 equiv.) in the sequence was coupled using PyBOP (1.0 equiv.), HOBt (1.1 equiv.), DIPEA (4.0 equiv.) in DMF (5 mL) under argon for 2 h. For final cleavage, resin-bound peptide analogues were suspended in 95/2.5/2.5 TFA/TIPS/H_2_O with shaking for 2–3 h. The cleaved resin was filtered, and the crude solution was concentrated *in vacuo*. Cold diethyl ether (2 × 5 mL) was added to triturate the crude residue. The diethyl ether was then decanted and centrifuged for 3 minutes at 13 000 rpm to pellet any residual peptide. The ether was removed, and the peptide pellet was then dried re-dissolved in 0.1% aqueous TFA. The peptides were purified with an Agilent C18 RP-HPLC column (100 Å, 5 μm, 250 mm, 4.6 mm), with an eluent system consisting of 0.1% aqueous TFA (solvent A) and 0.1% TFA in acetonitrile (solvent B). The analytical purification method used was: 0–3 min (10% B), 3–18 min (ramp 10–45% B), 18–25 min (ramp 45–100% B), 25–26.15 min (100% B), 26.15–27.25 min (ramp 100–10% B), and 27.25–36 min (10% B). The HPLC fractions were pooled and isolated as a white solid after lyophilization.

#### Synthesis of NMeLeu13A2, CbzPEG_6_–NMe17A2 and BMS-986224

The following analogues were synthesized according to previously reported literature methods.^14,26^

### ApelinR radioligand binding experiment

Crude membrane preparations from CHO cells stably expressing the wild-type human apelinR fused at its C-terminal part with EGFP (enhanced green fluorescent protein; clone G3a), were prepared as previously described.^42^ Membrane preparations were incubated for 3 h at 20 °C with 0.2 nM of the radioligand, [^125^I]-(Nle^11^, Tyr^13^) apelin-13 (radioiodinated in the laboratory on the tyrosine residue in position 13) in binding buffer (50 mM Hepes pH 7.5, 5 mM MgCl_2_, 1% bovine serum albumin) alone or in the presence of increasing concentrations of the various compounds (from 1 pM to 1 μM). The reaction was stopped with cold 10 mM Hepes pH 7.5, 1 mM MgCl_2_ buffer and filtered on Whatman GF/C filters. After washing the filters, radioactivity was measured using a Wallac gamma counter.

### Experimental animals

Wild-type (WT) mice with a C57BL/6 background were bred for this experiment as previously described.^2,11^ All mice were aged 12–16 weeks during the experiment. All animal experiments complied with the Canadian Council on Animal Care guidelines. The animal protocols were adopted, and the Animal Care and Use Committee reviewed and approved them at the University of Alberta.

### Blood pressure measurement and response to apelin analogues and small molecule agonist

The blood pressure measurement and apelin injection method has been described in our previous research.^43^ During the blood pressure testing, mice aged 12–14 weeks were anesthetized with 1.5% isoflurane in oxygen and monitored throughout the experiment. The blood pressure was measured using a PV loop catheter (model 1.2F, Transonic) calculated into the left carotid artery. The monitor system (LabScribe 2.0. Scisense) recorded aortic blood pressure and heart rate. After a 20-minute baseline measurement, CbzPEG_6_–NMeLeu17A2, NMeLeu13A2, RI13, RI13A2, RI17, RI17A2, and BMS-986224 (0.5 μmol k^−1^ body weight) were diluted in saline and consistently infused for 5 min *via* the right jugular vein. For BMS-986224, 0.1% DMSO was added to saline to help with dilution. Blood pressure was continuously recorded for 60 minutes post-infusion.

### *Ex vivo* heart perfusion with apelin analogues and small molecule agonist

Langendorff heart perfusion has been described previously to study the recovery of contractile function in ischemia–reperfusion injury.^2,11^ Mice aged 12–14 weeks were heparinized and euthanized with a ketamine/xylazine cocktail (10% ketamine with 5% xylazine in saline) before cannulation. The hearts were then mounted on the Langendorff system at consistent pressure of 80 mmHg and perfused with modified Krebs buffer (3.2 mmol L^−1^ KCl, 116 mmol L^−1^ NaCl, 1.2 mmol L^−1^ MgSO_4_, 2.0 mmol L^−1^ CaCl_2_, 25 mmol L^−1^ NaHCO_3_, 1.2 mmol L^−1^ KH_2_PO_4_, 11 mmol L^−1^d-glucose, 0.5 mmol L^−1^ EDTA and 2 mmol L^−1^ pyruvate) which incubated at 37 °C. The buffer was bubbled with carbogen (5% CO_2_ and 95% O_2_) to maintain the pH at 7.4. After removing the left atrium, a water-filling balloon was inserted into the left ventricle to measure pressure, which was recorded by the PowerLab system (AD Instrument, Australia). After stabilization of pressure, a 10-minute baseline was recorded, followed by 30 minutes of ischemia and 40 minutes of reperfusion. NMe17A2, NMeLeu13A2, BMS-986224, or placebo (1 μmol L^−1^) were administered during the first 10 minutes of reperfusion. 0.1% DMSO was added to improve the solubility of BMS-986224.

### Cell culture preparation for biased signalling assays

HEK293 clonal cell line (HEK293SL cells), hereafter referred as HEK293 cells, were a gift from S. Laporte (McGill University, Montreal, Quebec, Canada) and previously described.^44^ Cells were cultured in DMEM medium supplemented with 10% newborn calf serum iron fortified (NCS) and 1% of antibiotics (100 U mL^−1^ penicillin and 100 μg mL^−1^ streptomycin). Cells were passaged weekly and incubated at 37 °C in a humidified atmosphere with 5% CO_2_ and checked for mycoplasma contamination.

### Bioluminescence resonance energy transfer (BRET) measurement

Forty-eight hours before the experiments, 1 μg of total DNA (adjusted with salmon sperm DNA) was used to transfect 3.5 × 10^5^ cells per mL using linear polyethylenimine (PEI, 1 mg mL^−1^) diluted in NaCl (150 mM pH 7.0) as a transfecting agent (3 : 1 PEI/DNA ratio). Cells were immediately seeded (3.5 × 10^4^ cells per well) in 96-well white microplates (Greiner Bio one). Cells were maintained in culture for the next 48 h and BRET experiments carried out. Enhanced bystander BRET (ebBRET) was used to monitor the activation of Gαi1 and Gαi2 proteins, as well as β-arrestin 1 and 2 recruitment to the plasma membrane. Gαi proteins activation was followed using the selective-Gi/o effector Rap1GAP-RlucII and rGFP-CAAX along with the human Gαi1 or Gαi2 subunits and apelinR.^45^ β-arrestin recruitment to the plasma membrane was determined using β-arrestin1-RlucII or β-arrestin2-RlucII with rGFP-CAAX and apelinR.^44^ The day of the experiment, cells were washed with phosphate-buffered saline (PBS) and incubated in Tyrode Hepes buffer (137 mM NaCl, 0.9 mM KCl, 1 mM MgCl_2_, 11.9 mM NaHCO_3_, 3.6 mM NaH_2_PO_4_, 25 mM HEPES, 5.5 mM d-glucose and 1 mM CaCl_2_, pH 7.4) for 1 h at 37 °C. Cells were then treated with increasing concentrations of compounds for 10 min at 37 °C. The luciferase substrate Prolume purple (1 μM, NanoLight Technologies) was added during the last 6 min before the reading. BRET measurements were performed on a Tecan spark multimode microplate reader (Männedorf, Switzerland) using BRET^2^ setting: wavelength between 360 and 440 nm (donor) and, 505 and 575 nm (acceptor) for detecting the RlucII (donor) and rGFP (acceptor) light emissions, respectively. BRET signal (BRET^2^) was determined by calculating the ratio of the light intensity emitted by the acceptor over the light intensity emitted by the donor. The agonist-promoted BRET signal (ΔBRET) was calculated as the difference in BRET recorded from cells treated with compound and cells treated with vehicle before normalization in percentage of the maximal response elicited by the reference compound apelin-17. The data were analyzed in GraphPad prism 10.4.1 using “log(agonist) *vs.* response -- variable slope (four parameters)” and data were presented as mean ± SEM of at 5–6 independent experiments performed in simplicate.

### Western blotting analysis

Protein expression was assessed by Western blot analysis. Left ventricular tissue samples from mice in the Langendorff protocol were homogenized in RIPA buffer supplemented with protease and phosphatase inhibitors (Thermo Fisher Scientific, Waltham, MA, USA). Lysates were cleared by centrifugation at 12 000 × *g* for 15 min at 4 °C, and protein concentration was determined by the BCA assay (Pierce, Thermo Fisher Scientific, Waltham, MA, USA). Equal amounts of protein (20 μg per lane) were separated on 10% SDS-polyacrylamide gels and transferred to polyvinylidene difluoride (PVDF) membranes (Immobilon-P, MilliporeSigma, Burlington, MA, USA). Membranes were blocked for 1 h at room temperature in 5% bovine serum albumin (BSA) prepared in Tris-buffered saline with 0.1% Tween-20 (TBST) and incubated overnight at 4 °C with the following primary antibodies (all from Cell Signaling Technology, Danvers, MA, USA): phospho-Akt (Ser473; #4060), total Akt (#2920), phospho-ERK1/2 (Thr202/Tyr204; #9101), and total ERK1/2 (#4696), each diluted 1 : 1000 in blocking buffer. After washing with TBST, membranes were incubated with horseradish peroxidase (HRP)-conjugated anti-rabbit IgG secondary antibody (1 : 5000; Cell Signaling Technology, Danvers, MA, USA) for 1 h at room temperature. Protein bands were visualized using enhanced chemiluminescence (ECL, Bio-Rad, Hercules, CA, USA) and imaged with a ChemiDoc XRS+ system (Bio-Rad, Hercules, CA, USA). Band intensities were quantified using ImageJ software (National Institutes of Health, Bethesda, MD, USA), and phosphorylated protein levels were normalized to their corresponding total protein.

## Author contributions

AW, CF, KF, GYO, JCV conceptualization; AW, KF, CF, BH synthesis of peptides and small molecules; AZ physiological assays; CA BRET assays; XI radioligand displacement assays; DN Western blot assays; AW, KF, AZ, DN, CF, XI, CA investigation, analysis, and validation; AW, CF, AZ, DN, XI, CA writing – initial draft; AW, CF, AZ, DN, KF, XI, CA, GYO, BH, CLC, MB, JV writing – review & editing.

## Conflicts of interest

There are no conflicts to declare.

## Supplementary Material

MD-OLF-D5MD00985E-s001

## Data Availability

All data are contained within the article and in the supplementary information (SI). Supplementary information is available. See DOI: https://doi.org/10.1039/d5md00985e.
