# Supplementary material for: Screening apelin analogues and a small molecule agonist as effective cardiovascular therapeutics against reperfusion injury
Source: RSC Med Chem. 2025 Dec 19;17(1):236–44. doi: 10.1039/d5md00985e (PMC12715805; doi:10.1039/d5md00985e)
Supplement: MD-017-D5MD00985E-s001 [file MD-017-D5MD00985E-s001.pdf]

Supplementary Information (SI) for RSC Medicinal Chemistry.
This journal is © The Royal Society of Chemistry 2025

Screening apelin analogues and a small molecule agonist as effective cardiovascular therapeutics against reperfusion injury

Anjalee Wijewardane[a], Kleinberg X. Fernandez[a], Anran Zhang[bc], Conrad Fischer[a], Dineth P. Nagahawatta[bc], Xavier Iturrioz[e], Charlotte Avet[f], Bethan L. Heard[a], Catherine Llorens-Cortes[d,e], Gavin Y. Oudit[bc], Michel Bouvier[f], John C. Vederas[a]

Table of Contents

1. Chemical synthesis and structures of RI apelin analogues 2

a. RI-13 2

b. RI-13A2 2

c. RI-17 3

d. RI-17A2 3

2. Radioligand displacement assays of RI apelin analogues 4

3. Effects of APJ agonists on blood pressure 5

4. Biased signaling profile values for tested APJ ligands 6

1. **Chemical synthesis and structures of RI apelin analogues**

a. RI-13

Peptide RI-3 was synthesized following the general SPPS elongation procedure introducing amino acids in the following order: Fmoc-D-Arg(Pbf)-OH, Fmoc-D-Pro-OH, Fmoc-d-Arg(Pbf)-OH, Fmoc-D-Leu-OH, Fmoc-D-Ser(tBu)-OH, Fmoc-D-His(Trt)-OH, Fmoc-D-Lys(Boc)-OH, Fmoc-Gly-OH, Fmoc-D-Pro-OH, Fmoc-D-Met-OH, Fmoc-D-Pro-OH, and Fmoc-D-Phe-OH, with end-capping after each step. After the final N-terminal deprotection, the peptide was then cleaved from the resin and purified using the HPLC general method and an Agilent C18 RP-HPLC column (100 Å, 5 µm, 250 mm, 4.6 mm). The desired peptide was isolated as a white solid after lyophilization (6 mg, 12%). The desired peptide was isolated as a white solid after lyophilization (6 mg, 12%). Monoisotopic mass calculated for $C_{69}H_{111}N_{23}O_{16}S$ 775.9223, found (FTICR-ESI-MS) 775.9216 $[M + 2H]^{2+}$.

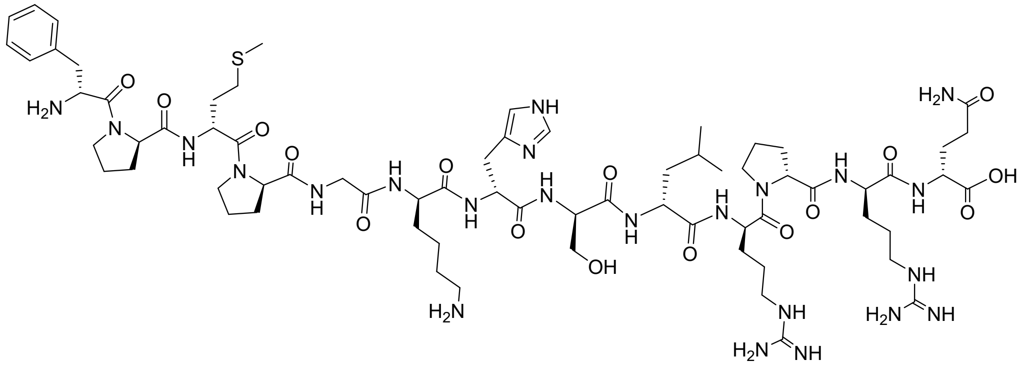

Fig. S1(a). Chemical structure of RI-13

b. RI-13A2

Peptide RI-13A2 was synthesized following the general SPPS elongation procedure introducing amino acids in the following order: FmocD-Arg(Pbf)-OH, Fmoc-D-Pro-OH, Fmoc-D-Arg(Pbf)-OH, Fmoc-D-Leu-OH, Fmoc-D-Ser(tBu)- OH, Fmoc-D-His(Trt)-OH, Fmoc-D-Lys(Boc)-OH, Fmoc-Gly-OH, Fmoc-D-Pro-OH, Fmoc-DMet-OH, Fmoc-Aib-OH, and Fmoc-D-BrF-OH, with end-capping after each step. After the final N-terminal deprotection, the peptide was then cleaved from the resin and purified using the HPLC general method and an Agilent C18 RP-HPLC column (100 Å, 5 µm, 250 mm, 4.6 mm). The desired peptide was isolated as a white solid after lyophilization (8 mg, 16%). Monoisotopic mass calculated for $C_{69}H_{112}BrN_{23}O_{16}$ 799.8993, found (FTICR-ESI-MS) 799.8981 $[M +2H]^{2+}$.

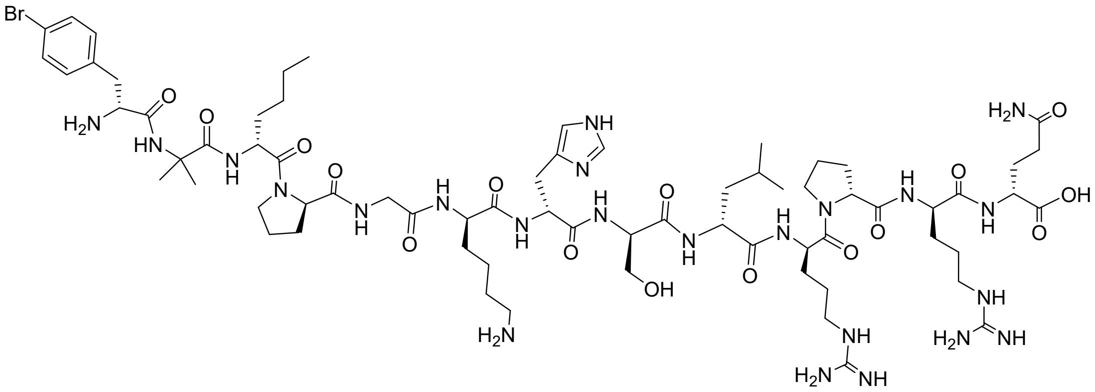

Fig. S1(b). Chemical structure of RI-13A2

c. RI-17

Peptide RI-17 was synthesized following the general SPPS elongation procedure introducing amino acids in the following order: FmocD-Phe-OH, Fmoc-D-Arg(Pbf)-OH, Fmoc-D-Arg(Pbf)-OH, Fmoc-D-Gln(Trt)-OH, Fmoc-DArg(Pbf)-OH, Fmoc-D-Pro-OH, Fmoc-D-Arg(Pbf)-OH, Fmoc-D-Leu-OH, Fmoc-D-Ser(tBu)-OH, Fmoc-D-His(Trt)-OH, Fmoc-D-Lys(Boc)-OH, Fmoc-Gly-OH, Fmoc-D-Pro-OH, Fmoc-D-MetOH, Fmoc-D-Pro-OH, and Fmoc-D-Phe-OH, with end-capping after each step. After the final N terminal deprotection, the peptide was then cleaved from the resin and purified using the HPLC general method and an Agilent C18 RP-HPLC column (100 Å, 5 µm, 250 mm, 4.6 mm). The desired peptide was isolated as a white solid after lyophilization (15 mg, 30%). Monoisotopic mass calculated for $C_{96}H_{156}N_{34}O_{20}S$ 1069.6051, found (FTICR-ESI-MS) 1069.605 $[M + 2H]^{2+}$.

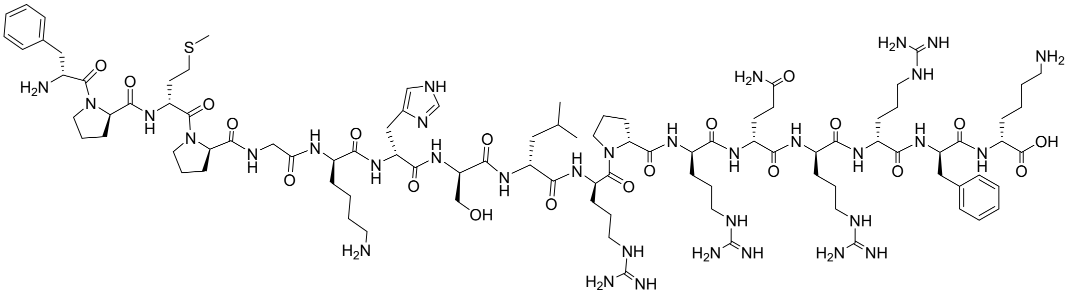

Fig. S1(c). Chemical structure of RI-17

d. RI-17A2

Peptide RI-17 was synthesized following the general SPPS elongation procedure introducing amino acids in the following order: FmocD-Phe-OH, Fmoc-D-Arg(Pbf)-OH, Fmoc-D-Arg(Pbf)-OH, Fmoc-D-Gln(Trt)-OH, Fmoc-D-Arg(Pbf)-OH, Fmoc-D-Pro-OH, Fmoc-D-Arg(Pbf)-OH, Fmoc-D-Leu-OH, Fmoc-D-Ser(tBu)-OH, Fmoc-D-His(Trt)-OH, Fmoc-D-Lys(Boc)-OH, Fmoc-Gly-OH, Fmoc-D-Pro-OH, Fmoc-D-Nle-OH, Fmoc-Aib-OH, and Fmoc-D-BrF-OH, with end-capping after each step. After the final N-terminal deprotection, the peptide was then cleaved from the resin and purified using the HPLC general method and an Agilent C18 RP-HPLC column (100 Å, 5 µm, 250 mm, 4.6 mm).

The desired peptide was isolated as a white solid after lyophilization (12 mg, 24%). Monoisotopic mass calculated for $C_{96}H_{157}BrN_{34}O_{20}$ 1093.5821, found (FTICR-ESI-MS) 1093.5839 $[M + 2H]^{2+}$.

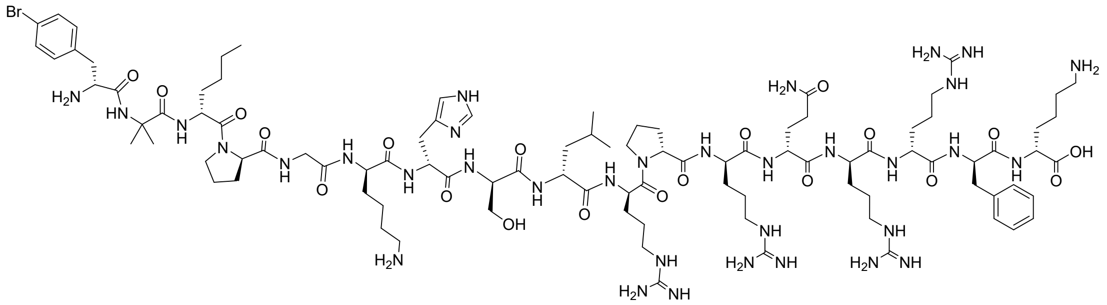

Fig. S1(d). Chemical structure of RI-17A2

*All RI peptides were obtained in 90-95% purity by HPLC. The trace impurities were identified as truncated, inactive peptides.

2. Radioligand displacement assays of RI apelin analogues

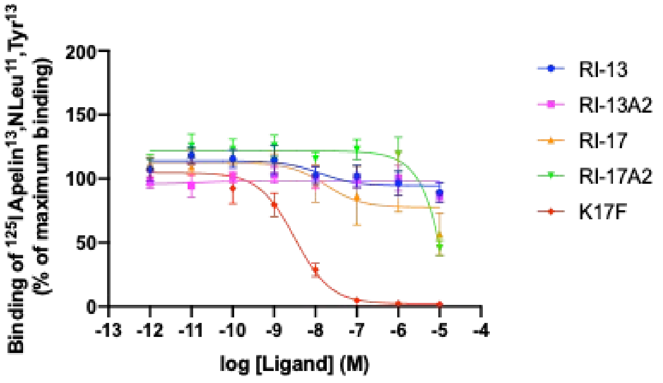

APJ receptor agonist	IC_{50} (nM)
apelin-17	2.46 ± 1.31 (7)
RI-13	n.d.
RI-13A2	>10^{-6} M (7)
RI-17	>10^{-6} M (7)
RI-17A2	>10^{-6} M (7)

Figure S2. Competition binding curves of different apelin receptor ligands. Membranes of CHO cells stably expressing the hApelinR were incubated with 0.2 nM of radiolabeled [125I]-Apelin-13 (Apelin 13, Nle11, Tyr13) in the presence of increasing concentrations of each ligand (from 1 pM to 10 µM). Data are expressed as a percentage of maximal binding of [125I]-Apelin-13 in the absence of any unlabeled ligand. Graphic is a compilation of 4 independent experiments performed in duplicate. Error bars represent the SE.

3. Effects of APJ agonists on blood pressure

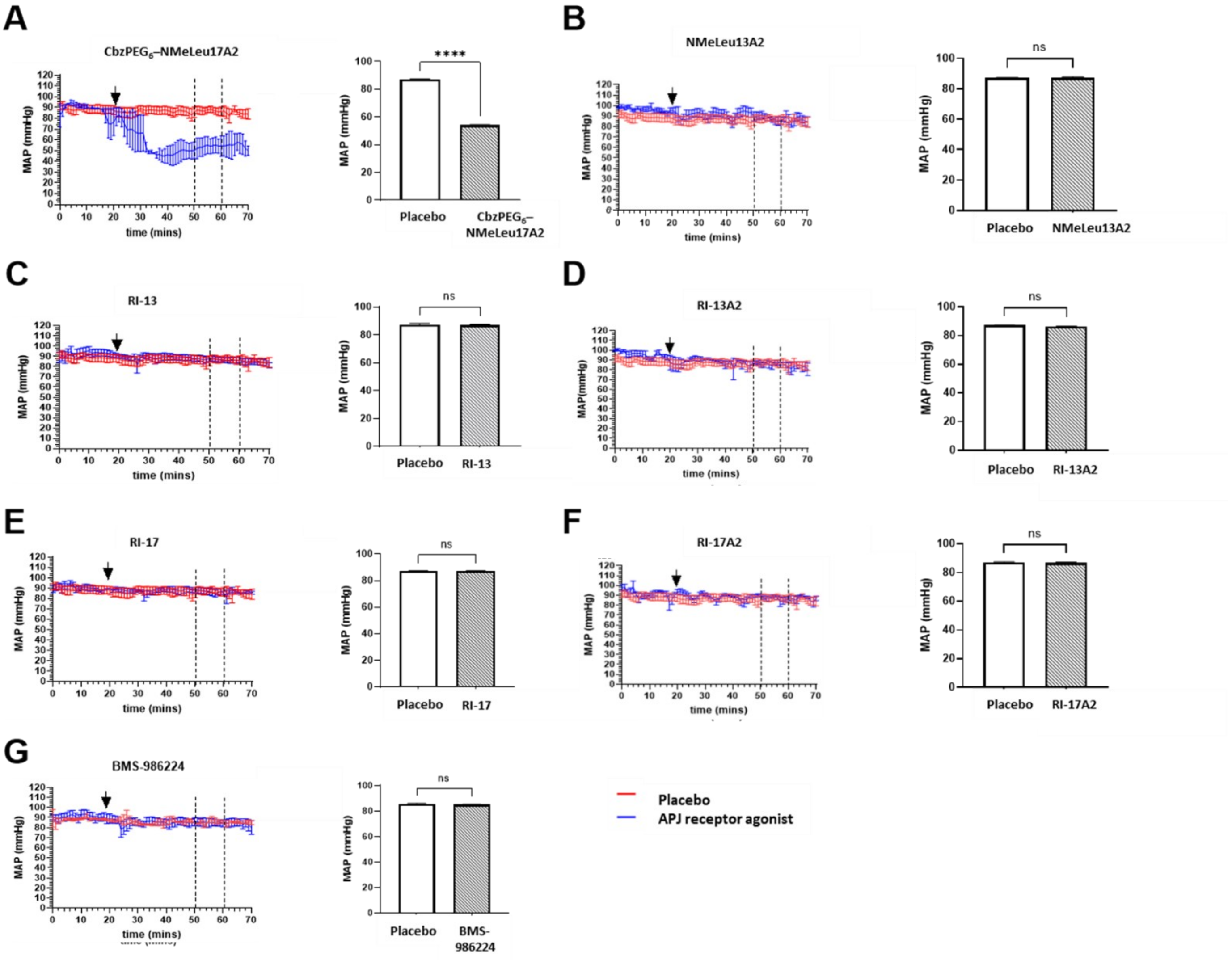

Figure S3. Different effects of apelin analog and receptor agonist on blood pressure and ischemia-reperfusion injury. A) the effect of $CbzPEG_6$–NMeLeu17A2 (n=3) on mean arterial blood pressure (MAP), B) the effect of apelin 13 analog, NMeLeu13A2, on MAP (n=4). The effect of apelin 13 analog reverse enantiomers, C) RI13 (n=4) and D) RI13A2 (n=4), on MAP. The effect of apelin 17 reverse enantiomers, E) RI17 (n=4) and F) RI17A2 (n=4) on MAP. G) The effect of small molecule apelin receptor agonist BMS-986224 on blood pressure (n=4). The MAP recording between 50 and 60 minutes has been averaged and compared with their controls (placebo (n=4)). The vehicle for BMS-986224 is 0.1% DMSO (n=3). The arrow indicates the time when analogs were introduced. Values mean ±SEM and ****$P<0.0001$, compared to the control group.

4. Biased signaling profile values for tested APJ ligands

Table S1. BRET-detected logEC_{50} values for individual activation pathways

	**Gαi1 activation log EC_{50}**	**Gαi2 activation log EC_{50}**	**β-arr1 recruitment log EC_{50}**	**β-arr2 recruitment log EC_{50}**
**AMG-986**	10.56 ± 0.07	10.67 ± 0.04	8.96 ± 0.04	9.00 ± 0.03
**BMS-986224**	9.99 ± 0.05	9.96 ± 0.04	7.97 ± 0.04	8.05 ± 0.02
**pyr-apelin-13**	9.78 ± 0.06	9.75 ± 0.04	7.74 ± 0.02	7.76 ± 0.01
**apelin-17**	10.69 ± 0.10	10.78 ± 0.02	8.77 ± 0.02	8.74 ± 0.03
**NMe13A2**	10.47 ± 0.06	10.66 ± 0.04	8.91 ± 0.03	8.94 ± 0.03
**Cbz-PEG_6-NMeLeu17A2**	10.52 ± 0.08	10.73 ± 0.04	9.04 ± 0.03	8.81 ± 0.07
**apelin 8-mer C-terminal**	6.31 ± 0.05	6.46 ± 0.03	n.d.	n.d.
**apelin 9-mer N-terminal**	n.d.	n.d.	-	-
**apelin-5-mer C-terminal**	5.97 ± 0.06	5.94 ± 0.04	-	-
